# Supplementary material for: A strict experimental test of macroscopic realism in a superconducting flux qubit
Source: Nat Commun. 2016 Nov 4;7:13253. doi: 10.1038/ncomms13253 (PMC5097155; doi:10.1038/ncomms13253)
Supplement: Supplementary Information — Supplementary Figures 1, Supplementary Table 1, Supplementary Notes 1-3 and Supplementary References. [file ncomms13253-s1.pdf]

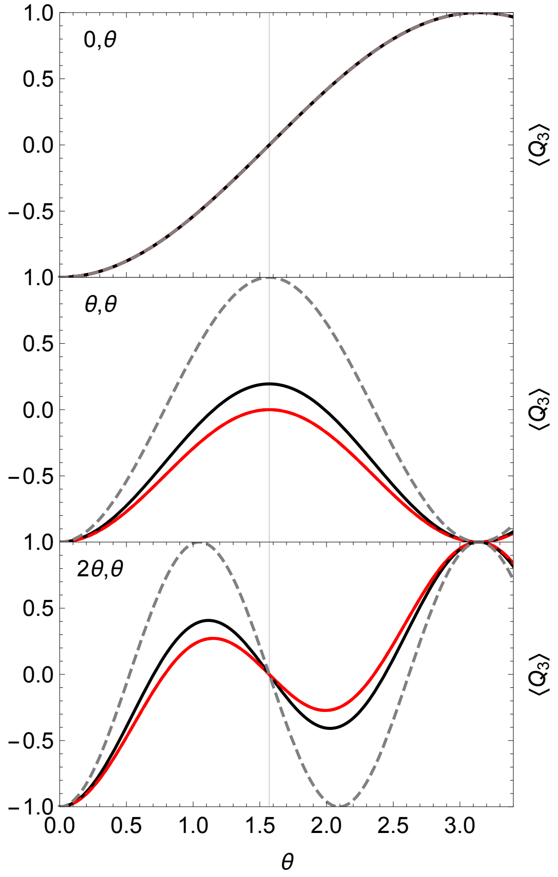

Supplementary Figure 1: Theoretical predictions shown in equation (12), prodced by a simple quantum-mechanical model. Red (black) curves correspond to the presence (absence) of the measurement pulse $\mathcal{O}$, intervening between two rotations by angles θ_1, θ_2. Grey, dashed curves relate to the case of no measurement pulse and an infinite T_2 time. Compare with Figure 3 of the main text.

Q_1	Q_2	inequality	implication
1	-1	LG2′	$d \leq 0$
1	-1	LG1′	$d \geq 0$
1	1	LG3′	$d \geq 0$
1	1	LG4′	$d \leq 0$
-1	1	LG2′	$d \leq 0$
-1	1	LG1′	$d \geq 0$
-1	-1	LG3′	$d \leq 0$
-1	-1	LG4′	$d \geq 0$

Supplementary Table 1: **Logical implications:** By fixing the values of Q_1 and Q_2 and by using multiple Leggett-Garg inequalities, one can derive the non-disturbance condition $d = 0$.

Supplementary Note 1: Three types of measurement invasiveness, and the logical relation of NDC to LGI

In the main text we argued directly from NIM to the NDC. It is possible however, to argue from

$$\text{LGI}: \qquad \langle Q_1Q_2\rangle_{\bar{3}} + \langle Q_1Q_3\rangle_{\bar{2}} + \langle Q_2Q_3\rangle_{\bar{1}} \geq -1. \tag{1}$$

to the NDC. It is clear that a slight modification from LGI to

$$\text{LG1}': \qquad \langle Q_1Q_2\rangle_{G} + \langle Q_1Q_3\rangle_{\bar{2}} + \langle Q_2Q_3\rangle_{\text{G}} \geq -1; \tag{2}$$

also follows via NIM, and captures all of the essential features of the LGI. The advantage is that now only two ensembles need be discussed. One should in principle check for the disturbance of any measurement which appears in some ensembles and not in others during the course of the test of macrorealism. With the full machinery of LGI in place, this would involve testing

$$d_c^{\text{I}} := \langle Q_2Q_3\rangle_{\text{G}} - \langle Q_2Q_3\rangle_{\bar{1}} \tag{3}$$

$$d_c^{\text{II}} := \langle Q_1Q_3\rangle_{\text{G}} - \langle Q_1Q_3\rangle_{\bar{2}} \tag{4}$$

$$d_c^{\text{III}} := \langle Q_1Q_2\rangle_{\text{G}} - \langle Q_1Q_2\rangle_{\bar{3}} \tag{5}$$

in control experiments, and then looking for satisfaction or violation of

$$\langle Q_1Q_2\rangle_{\bar{3}} + \langle Q_1Q_3\rangle_{\bar{2}} + \langle Q_2Q_3\rangle_{\bar{1}} \geq -1 - d_c^{\text{I}} - d_c^{\text{II}} - d_c^{\text{III}}. \tag{6}$$

The assumption that $d_c^{\text{III}} = 0$ is often motivated by appeals to the unlikelihood of retrocausality (called Induction by Leggett): $d_c^{\text{I}} = 0$ however, has been often mistakenly assumed in the literature despite its negation being quite plausible in a general theory. This is perhaps because it is true

according to quantum mechanics: any influence propagating from t_1 to t_3 is completely screened off by a projective measurement at t_2. For a fuller discussion of these issues, see Ref.[1]. Our simplified protocol however, negates the need to test for d_c^{I} or d_c^{III} since we never use ensembles $\bar{1}$ or $\bar{3}$. In reducing the number of distinct ensembles from four (G, $\bar{3}, \bar{2}, \bar{1}$) to two (G, $\bar{2}$), we need only consider the possible effects of a measurement (or not) at t_2.

The issue of experimentally determining the disturbance of measurements was discussed in a proposal from Wilde and Mizel[2]. They call a measurement ϵ-adroit if the total change in joint probability for outcomes of measurements made before and after the measurement is upper-bounded by ϵ, when the joint probability is conditioned on either i) performing the measurement or ii) not performing the measurement. It is in this spirit that we pursue the experimental determination of measurement disturbance – previously only *a priori* arguments for $d_c = 0$ (or its equivalent) have been employed.

To see the logical connection between LGI and NDC, consider that under MRPS there are only four possible values for Q_1 and Q_2. For each combination, a pair of inequalities together imply the non disturbance condition. Take the $Q_1 = 1 = -Q_2$ case as an example. The LG1$'$ condition (2) becomes $d \geq 0$ and a partner inequality

$$\mathrm{LG2}': \qquad \langle Q_1 Q_2 \rangle_{\mathrm{G}} - \langle Q_1 Q_3 \rangle_{\bar{2}} - \langle Q_2 Q_3 \rangle_{\mathrm{G}} \geq -1, \tag{7}$$

obtained by changing the sign of two correlators, becomes $d \leq 0$. These two inequalities together

imply $d = 0$. In formal notation ($\wedge$ for logical conjunction, $\rightarrow$ for implication, $\neg$ for negation)

$$(Q_1 = 1) \wedge (Q2 = -1) \wedge (\mathrm{LG1}') \rightarrow d \geq 0 \tag{8}$$

$$(Q_1 = 1) \wedge (Q2 = -1) \wedge (\mathrm{LG2}') \rightarrow d \leq 0 \tag{9}$$

$$(d \geq 0) \wedge (d \leq 0) \rightarrow d = 0 \quad . \tag{10}$$

Similar arguments can be made for the other three combinations, which make use of another pair of inequalities LG3$'$: $-\langle Q_1 Q_2 \rangle_{\mathrm{G}} + \langle Q_1 Q_3 \rangle_{\bar{2}} - \langle Q_2 Q_3 \rangle_{\mathrm{G}} \geq -1$ and LG4$'$: $-\langle Q_1 Q_2 \rangle_{\mathrm{G}} - \langle Q_1 Q_3 \rangle_{\bar{2}} + \langle Q_2 Q_3 \rangle_{\mathrm{G}} \geq -1$.

The eight logical implications required to secure all possibilities are shown in Supplementary Table 1. Given a violation of NDC, one can always choose a fixed assignment of ± 1 values to Q_1 and Q_2 such that at least one simplified LG inequality is duly violated:

$$\neg \mathrm{NDC} \rightarrow (\neg \mathrm{LG1}') \vee (\neg \mathrm{LG2}') \vee (\neg \mathrm{LG3}') \vee (\neg \mathrm{LG4}'). \tag{11}$$

Furthermore, given a fixed value assignment of Q_1 and Q_2, the sign of the violation of the NDC will inform as to which one of a pair of LG inequalities is violated.

Supplementary Note 2: Ideal quantum mechanical predictions for Figure 3 of the main text

Although our refutation of macroscopic realism does not require that quantum mechanical predictions are reproduced, for completeness we show that the results in Figure 3 of the main text are captured by a simple quantum mechanical model. Our qubit begins in the ground state $|g\rangle$, and then is subject to a pseudo-spin rotation through angle θ_1. Next, it either experiences environ-

mental dephasing for a fixed period $t \sim 18$ ns, or a measurement pulse $\mathcal{O}$ which causes complete dephasing. It is then subject to another pseudo-spin rotation through angle θ_2 before being subject to a measurement of $Q_3 = |e\rangle\langle e| - |g\rangle\langle g|$. The expectation value is

$$
\begin{aligned}
\langle Q_3 \rangle_{\mathrm{G}} &= -\cos\theta_1 \cos\theta_2 \\
\langle Q_3 \rangle_{\bar{2}} &= -\cos\theta_1 \cos\theta_2 + e^{-t/T_2} \sin\theta_1 \sin\theta_2
\end{aligned}
\qquad (12)
$$

where as before the subscript G ($\bar{2}$) denotes that the operation $\mathcal{O}$ was (was not) performed, and $T_2 \sim 10$ns is the coherence time. These curves are plotted in Supplementary Figure 1: although our data are in good qualitative agreement, a more sophisticated model that takes account of asymmetric measurement visibility would match the experimental data even more closely.

Supplementary Note 3: Macroscopicity

To what extent is it legitimate to speak of the two states between which our measuring device discriminates as "macroscopically distinct"? More generally, do the two states which characteristically occur in a flux qubit, in particular the two flux eigenstates corresponding respectively to clockwise and counterclockwise circulating currents, deserve this description? This question has been subjected to considerable discussion in the recent literature, so we need to address it briefly. A point which needs to be made forcefully at the start of any such discussion is that the notion of "macroscopic distinctness" or more generally of "macroscopicity" is not given a priori; it is *a matter of definition*, and the definition which one finds most useful or natural may well depend on the context in which one intends to apply it. This should be borne in mind when reading comparisons in the literature of very different kinds of physical system which use the authors' favorite figure of

merit. In our case, and more generally in the history of experiments on flux qubits, the motivation for trying to define a measure of “macroscopic distinctness” has been to try to quantify the instinctive feeling that we suspect most physicists as well as most laypersons have that while the idea that an electron faced with the choice of two separated slits simultaneously may not definitely choose either is bizarre but (at least in 2016) tolerable, the notion of a cat which is neither definitely alive nor definitely dead is still distinctly alarming. For a refutation of the widespread misconception that the problem is resolved by the phenomenon of decoherence, see Ref.[3]. Thus we would like to define some kind of measure of how far a given experiment has progressed along the axis from the world of electrons and atoms to the world of our own direct experience: a measure, if you like, of the degree of “Schrödinger’s-cattiness”.

In what may have been the first attempt in the literature to define such a figure of merit, one of us (AJL) introduced in effect two relevant concepts, that of “extensive difference” and of “disconnectivity”. The former, which is introduced explicitly in Ref.[4] is definable quite independently of any quantum-mechanical preconceptions; it is simply a measure of the difference in some extensive physical quantity in the two states which the measurement discriminates, normalized to some natural atomic-scale unit. (Of course, since by defining a sufficiently complicated extensive variable one could probably obtain an arbitrarily large value of the extensive difference, one needs to make some implicit assumption about the naturalness of the variable selected). The latter was introduced to distinguish between the various possible decompositions of the components of a superposition (having a certain extensive difference) which involve *collusion* to a greater or lesser extent[5]. Consider that the a large change of state of one particle alone might account for the exten-

sive difference, in contrast to the case (as is presumably so for Schrödinger's cat) where the total difference is contributed to by a large number of colluding constituents each changing by smaller 'intensive' difference. The disconnectivity should measure the number of particles that behave differently in the two branches of the superposition. As with entanglement measures, with which the disconnectivity shares an affinity, there are a variety of ways of doing so – an example is shown below.

With regard to the "extensive difference" figure of merit, the situation in respect of the present experiment seems unambiguous, at least provided we are prepared to accept the magnetic moment as a "legitimate" extensive variable and take the normalization as the Bohr magneton μ_B. Because in our experiment we use a persistent current of $I_p = 170$nA flowing around a loop of cross sectional area 7 μm^2, in principle we have an extensive difference in magnetic moment of approximately $2 \times 130,000\mu_B$. However, our states $|g\rangle$ and $|e\rangle$ are not states of definite flux (see Methods). We have instead an extensive difference of $0.7 \times 2 \times 130,000\mu_B$.

With regard to the "disconnectivity", the situation is more complicated. If one performs a detailed microscopic calculation of the number of electrons (each having a relatively high typical momentum) that must change state to take one persistent current state of the flux qubit to the other this number is given by the formula

$$\Delta N = \frac{6L}{4e\nu_f} I_p \tag{13}$$

as Korsbakken et al[6] show (L is the circumference of the loop, e is the electron charge and ν_f is the Fermi velocity). For our experiment, it takes a value of about 8. To give some intuitive meaning

to this number, consider (as one of us has shown[7]) that the corresponding figure for two states of the smallest dust particle visible with the unaided naked eye, one stationary and one moving over its diameter in a second, is $\sim$2.5. Both numbers increase if the respective extensive differences are shared out among the *composite* constituents which the fundamental constituents are bound into. The nucleons in the dust molecule reside within a nucleus, which has significantly lower average momentum. If the extensive difference is 'cashed out' in units of this lower momentum, around 160 nuclei would need to contribute. For the electrons in the flux qubit, the same argument using the lower momentum of a Cooper pair implies that a number of Cooper pairs several order of magnitudes higher than Korsbakken et. al's number of electrons (13) would need to be involved to explain the extensive difference[7]. The disconnectivity of our experiment could then become quite considerably larger than something ostensibly on the human scale.

The reader may wonder why we have not employed the definition of "macroscopicity" proposed in a recent paper by Nimmrichter and Hornberger (NH)[8], which judging by its citations seems to have been widely accepted as in some sense canonical. Of course, this figure is even smaller for our experiment than for some previous ones on flux qubits, which in turn, as shown by NH, are much less "macroscopic" by their measure than various experiments at the atomic or molecular level. The reason is that NH are interested in formulating a figure of merit for a *very specific class* of theories alternative to QM, namely those which introduce some small corrections to the theory which are amplified as one goes from the atomic to the everyday level, and which in addition are constrained by their postulates (i)-(iv)(the prototype is that of the objective collapse theories from Ghirardi, Rimini and Weber[9] and from Pearle[10]). While we agree that the NH crite-

rion is very useful for the quantification of these corrections, we believe that in the context of our enterprise of "building Schrödinger's Cat in the laboratory" it is not always relevant, for at least two reasons: Firstly the constraints imposed by NH seem to us unduly restrictive; for example, relaxing their eqn.(2) to allow coupling not just to mechanical but to electromagnetic variables might yield a much larger figure of merit for flux qubits. In short, the NH measure presupposes a modification to quantum theory primarily concerned with mass, and this fact may imply that certain types of states we might wish to call unambiguously macroscopic are not so certified by the theory. We note that in Milburn's theory of intrinsic decoherence[11] "...the rate of diagonalization [collapse] depends on the square of the energy separation of the superposed states". This is but one concrete example of the different possible choices of macroscopicity scale. Secondly and most importantly, however, a future theory which allows definite outcomes at the level of everyday life, and hence supersedes QM at that level, is likely to be at least as different in its fundamental concepts from QM as the latter is from classical physics; and just as it is impossible (or possible only with a vast amount of hindsight) to view the classical-quantum transition in terms of "minimal modifications" to classical physics, the same is likely to be true of a future conceptual revolution, if such should indeed occur, which overthrows QM itself. Thus we prefer to use a figure of merit for "macroscopicity" which is independent of QM considerations and better attuned to our common-sense notions of what distinguishes the Young's-slits and Schrödinger's-Cat (thought-) experiments. Of course, this does not exclude the possibility that other types of system such as optically levitated microspheres may in future yield larger values of this figure than that obtained here, or indeed attainable with any practical flux-qubit system.

Supplementary references

1. Yeh, M.-C. *Some theoretical considerations related to the experimental tests of a temporal Bell's inequality*. Ph.D. thesis, University of Illinois at Urbana-Champaign (2015).

2. Wilde, M. & Mizel, A. Addressing the Clumsiness Loophole in a Leggett-Garg Test of Macrorealism. *Foundations of Physics* **42**, 256–265 (2011).

3. Adler, S. L. Why decoherence has not solved the measurement problem: a response to P.W. Anderson. *Studies in History and Philosophy of Science Part B: Studies in History and Philosophy of Modern Physics* **34**, 135 – 142 (2003). URL http://www.sciencedirect.com/science/article/pii/S1355219802000862.

4. Leggett, A. J. Testing the limits of quantum mechanics: motivation, state of play, prospects. *Journal of Physics: Condensed Matter* **14**, R415–R451 (2002).

5. Leggett, A. J. Macroscopic quantum systems and the quantum theory of measurement. *Progress of Theoretical Physics Supplement* **69**, 80–100 (1980). URL http://ptp.ipap.jp/link?PTPS/69/80/.

6. Korsbakken, J. I., Wilhelm, F. K. & Whaley, K. B. The size of macroscopic superposition states in flux qubits. *EPL (Europhysics Letters)* **89**, 30003 (2010). URL http://stacks.iop.org/0295-5075/89/i=3/a=30003.

7. Leggett, A. J. Note on the 'size' of Schrödinger cats (2016). URL http://arxiv.org/abs/1603.03992v1.

8. Nimmrichter, S. & Hornberger, K. Macroscopicity of mechanical quantum superposition states. *Phys. Rev. Lett.* **110**, 160403 (2013). URL `http://link.aps.org/doi/10.1103/PhysRevLett.110.160403`.

9. Ghirardi, G. C., Rimini, A. & Weber, T. Unified dynamics for microscopic and macroscopic systems. *Phys. Rev. D* **34**, 470–491 (1986).

10. Pearle, P. Combining stochastic dynamical state-vector reduction with spontaneous localization. *Phys. Rev. A* **39**, 2277–2289 (1989). URL `http://link.aps.org/doi/10.1103/PhysRevA.39.2277`.

11. Milburn, G. J. Intrinsic decoherence in quantum mechanics. *Phys. Rev. A* **44**, 5401–5406 (1991). URL `http://link.aps.org/doi/10.1103/PhysRevA.44.5401`.
